# Global gene expression profiling of ischemic preconditioning in the rat retina

**Published:** 2007-06-28

**Authors:** W. Kamphuis, F. Dijk, S. van Soest, A.A.B. Bergen

**Affiliations:** 1 Department of Ophthalmogenetics, Netherlands Institute for Neuroscience NIN-KNAW, Amsterdam, Netherlands; 2 Department of Cellular Quality Control, Netherlands Institute for Neuroscience NIN-KNAW, Amsterdam, Netherlands

## Abstract

**Purpose:**

To obtain and analyze the gene expression changes after ischemic preconditioning (IPC) in the rat retina.

**Methods:**

Ischemic damage to the inner retina can be prevented by a short, non-deleterious, ischemic insult of 5 min applied 24 h preceding a full ischemic insult of 60 min; a phenomenon termed tolerance or IPC. The time course of changes in gene expression after induction of IPC was assessed by 22K oligonucleotide microarrays, followed by real-time quantitative polymerase chain reaction (qPCR) validation. Functional pathways of interest were identified by Gene Ontology-term analysis.

**Results:**

Histology confirmed that IPC induction by 5 min of retinal ischemia results in a complete protection against the neurodegenerative effects of a 60 min ischemic period applied 24 or 48 h later. The microarray analysis revealed differential expression of 104 known genes at one or more time points between 1 h and 7 days after IPC. The group of altered genes contained a significant overrepresentation of genes involved in aminoacyl-tRNA synthetase activity (*Iars*, *Lars*, *Cars*, *Yars*, *Gars*, *Tars*), amino acid transport (*Slc3a2*, *Slc6a6*, *Slc7a1*, *Slc38a2*), regulation of transcription (including *Egr1*, *Egr4*, *Nr4a1*, *Nr4a3*, *c-fos*), and cell death (including *Anxa1*, *Trib3*). qPCR assays on cDNA of individual animals confirmed the microarray results.

**Conclusions:**

Endogenous neuroprotection, provoked by ischemic preconditioning is associated with changes in transcript levels of several functionally-related groups of genes. During the time window of effective protection, transcript levels of genes encoding for aminoacyl-tRNA synthetases and for amino acid transport are reduced. These changes suggest that a reduction of translational activity may play a significant role in preconditioning-mediated neuroprotection.

## Introduction

The gradual and selective loss of retinal ganglion cells by apoptosis underlies the progression of glaucoma and leads to a loss of vision. A variety of causal factors have been put forward that may bring about ganglion cell apoptosis. The relative contribution of these factors to the onset and progression of glaucoma however remains unknown. Hypoxia/ischemia are some of the factors implicated in glaucoma and several other retinal disease conditions such as diabetic retinopathy and central retinal artery occlusion [1-6]. The preferential loss of retinal ganglion cells in the glaucomatous retina corresponds to the pattern of neuronal degeneration through apoptosis after experimental ischemia [7,8]. Therefore, a better understanding of the molecular pathways triggered after experimental ischemia will help to identify factors that determine ganglion cell loss in glaucoma [9]. Of equal interest are intrinsic neuroprotective factors/pathways that enable retinal cells to cope with stressful conditions. The discovery of ischemic preconditioning (IPC, also termed tolerance) provides an ideal experimental opportunity to study these mechanisms. IPC is induced by a short 5 min period of ischemia which by itself does not lead to cell loss. When the retina is challenged by a full 60-min ischemic insult 24 h later, normally leading to the degeneration of cells in the inner retina, both a robust protection against the loss of cells and a preservation of a- and b-waves in the electroretinogram is observed [10,11]. IPC provides a transient protective effect lasting 24 to 72 h after the preconditioning stimulus and depends upon de novo protein synthesis [10,11]. The mechanism of IPC is not completely understood, but Hif1 transcription factor [12-14], Hsp27 (Hspb1) upregulation [10,14-16], stimulation of adenosine A1 and A2a receptors [13,17,18], protein kinase C activation [18], leukocyte rolling inhibition [19], inducible nitric oxide synthase (iNOS) [20], and the opening of mitochondrial potassium-dependent ATP (mKATP) channels [21] have been proposed as underlying factors (see for reviews [10,22]: ).

Microarray technology allows for a comprehensive screening of changes in the expression of genes in different physiological states. Microarray studies on IPC in rat spinal cord, rat hippocampus and mouse cortex have identified several genes that may be involved, including heat-shock proteins and transcription factors [13,23,24]. The aim of the current study is to document the molecular pathways that underlie IPC-induced neuroprotection in the retina.

## Methods

### Animals and anesthetics

Animal handling and experimental procedures were reviewed and approved by the ethical committee for animal care and use of the Royal Netherlands Academy for Sciences, acting in accordance with the European Community Council directive of 24 November 1986 (86/609/EEC) and the ARVO statement for the use of animals in Ophthalmic and Vision Research. The procedure to induce transient ischemia followed by reperfusion has been described in detail previously [25-27]. In short, adult male Wistar rats (Harlan, the Netherlands) weighing 200-300 g were anesthetized and mounted onto a stereotactic frame (Stoelting, Wood Dale, IL). A steel 30-gauge infusion needle (BD Biosciences, Alphen aan den Rijn, The Netherlands) connected to a saline reservoir was placed in the middle of the anterior chamber of the left eye. The reservoir was opened and lifted to 1.70 m. Ischemia was confirmed by the white anemic fundus reflex. After 60 min of ischemia, the reservoir was lowered and reperfusion started immediately. For IPC, 5 min of ischemia was applied and retinas were isolated after 1 h (n=6), 3 h (n=6), 6 h (n=5), 12 h (n=5), 24 h (n=5), 48 h (n=5), and 7 days (n=6). Animals were killed by an overdose of sodium-pentobarbital (Ceva Santa Animale, Maassluis, The Netherlands; 0.8 ml; 60 mg/ml) intraperitoneally. Sham-operated animals, in which a needle was inserted into the anterior chamber without elevating pressure, were included in each control group to evaluate the possibility that the process of cannulation itself induces differences between ischemic and control eyes.

### Histology

A separate group of animals was preconditioned by 5 min of ischemia, and following different intervals (24 h, 48 h, 7 days; n=3 each group) challenged by 60 min ischemia. After a delay of 7 days, the animals were sacrificed for histological assessment of inner plexiform layer thickness and somata counts in the ganglion cell layer [25].

### RNA isolation

The isolation of total RNA from the retina has been described in detail [28]. In short, the whole of the retina was isolated, frozen on dry ice and stored at -80 °C. Frozen retina was thawed in Trizol (Invitrogen, Breda, The Netherlands) and homogenized immediately. Total RNA was isolated following the manufacturer's instructions. Precipitated RNA was dissolved in 8 μl RNase-free water. The RNA yield was around 10 μg/retina (ND-1000 Spectrophotometer; NanoDrop Technologies, Wilmington, DE) and quality checks showed sharp ribosomal RNA peaks with minimal degradation (Agilent Technologies 2100 Bioanalyser, Amstelveen, The Netherlands).

### Experimental set-up and probe generation

Pooled total RNA samples were prepared corresponding to each of the time points after IPC and one additional pooled sample was propared from all contralateral control retinas (n=36). Each retinal sample contributed one μg of total RNA to the pooled sample. For first strand cDNA synthesis, 1.5 μg RNA of the pooled sample was primed with a T7 Oligo(dT) (Ambion, Nieuwerkerk a/d lJssel, The Netherlands) primer. Following second strand cDNA synthesis, in vitro transcription generated amino allyl-UTP labeled aRNA (Amino Allyl MessageAmp aRNA kit, Ambion, Nieuwerkerk a/d lJssel, The Netherlands) with an average yield of 40-60 μg. Electropherograms of the amplified aRNA showed a symmetrical length distribution with a peak around 1.5 kb, a maximum length of 5.5 kb, and no differences in size distribution between the different samples. The aRNA was coupled to Cy3 or Cy5 monoreactive dyes (Amersham, Eindhoven, The Netherlands) and purified from excess dye over a Chromaspin-30 column (Clontech, Westburg, Leusden, The Netherlands). Incorporation efficiency and yield were determined on a spectrophotometer (NanoDrop Technologies).

For microarray hybridization, a common reference design was used. aRNA of the control-group (30 μg) was labeled with Cy3. An aliquot of 1 μg served as the identical common reference present on all arrays and was hybridized simultaneously with 1 μg Cy5-labeled aRNA from one of the experimental or control groups.

### Microarrays

Oligonucleotide arrays were obtained from Agilent (Amstelveen, The Netherlands; 22K catalog rat array, 60-mer oligonucleotides, product number G4130A); the complete design can be found at Agilent or GEO-NCBI. These arrays are designed for the simultaneous hybridization of two aRNA samples labeled with Cy5 (treatment-group) and Cy3 (control). After normalization, the dye ratio represents the transcript ratio. On Agilent arrays, gene expression levels are monitored by a single carefully selected probe (termed feature) aimed to combine high sensitivity with the least variable ratio. This approach results in a low technical inter-chip variability [29,30]. In our experience, the technical reproducibility between arrays is indeed high: the outcome between two technical replicates with the same or reversed polarity shows a correlation around R^2^=0.77. Hybridization was carried out according to the protocols described by Agilent. For details see: Two-color microarray-based gene expression analysis at Agilent. After hybridization, the slides were washed for 5 min in 6x SSPE+0.005% N-lauroylsarcosine (Sigma-Aldrich, Zwijndrecht, The Netherlands) at room temperature, washed for 1 min in 0.06x SSPE+0.005% N-lauroylsarcosine, dipped in acetonitril (Sigma-Aldrich, Zwijndrecht, The Netherlands), and dried with a stream of N_2_.

### Data analysis

The array images were acquired using an Agilent microarray scanner set at 5 μm resolution. Images were loaded into Feature Extraction software (v8.5; Agilent) and combined with the latest array information (design-file) with default settings for all parameters including normalization. For details see feature extraction software user manual v8, at Agilent. This results in the signal strength of the treatment (Cy5) and control (Cy3) channels, the relationship between the two channels in terms of log ratio, the associated p-value and information concerning various quality control fields. The arrays studied had on average 22 technically flawed features (outliers; 0.1%). Images and MAGE files were uploaded to Rosetta Biosoftware Resolver® system, v5.0. Detailed information on the Rosetta Resolver system can be found at Rosetta Biosoftware (Rosetta Biosoftware, Seattle, WA), and in several published papers [31,32]. The complete list of all signatures, log_10_ ratios, log ratio errors, and p-values is also available as supplemental data (Appendix 1).

### Selection considerations

The Agilent and Rosetta systems determine differential expression not on simple constant fold-change thresholds but upon signal/noise ratios. The Feature Extraction software extends the accuracy of the data by propagating errors for each signal and background measured. Calculations using this propagated error and Rosetta's error model yield a log ratio error and p-value for the log ratio of each feature. The error around log ratios is signal intensity-dependent. The default additive and multiplicative parameters have been optimized for the Agilent microarrays (e.g., microarrays, protocols, scanner, and feature extraction algorithms). To select appropriate cut-off values, the following was considered: our study design used pooled-samples for the microarrays and must be considered as a fast screening tool to select features of interest, followed by gene ontology analysis and a series of confirmatory quantitative PCR (qPCRs) on the individual animals. Several published papers have shown a high correlation between the data from Agilent's oligo arrays and qPCR [33,34]. Based upon previously collected qPCR data on the transcript levels of 80 different genes in untreated and ischemia-treated rat retinas, the average biological variability of transcript levels was estimated to be 41±14% (coefficient of variation %±S.D.) [26,28]. The arrays present the average fold-change between 5 IPC-treated animals compared to 36 control retinas. Power calculations show that for transcript levels with variability of 55% (mean±SD), a >1.7 fold change will be declared as significantly regulated by qPCR assays (p<0.05; power 0.80). We therefore selected features with >1.7 fold change. Ranking the micro array data on fold-change, some >1.7 fold changes were found to be based on low intensity features just above background. The technical log ratio error, assigned by Feature Extraction software, was therefore added as a second criterion (p<0.01).

Preliminary experiments identified a significant dye bias in 19 features and these were removed from analysis. A set of 56 features were flagged as saturated in most of the arrays and these features were excluded from analysis. Furthermore, a set of 25 features (Appendix 1) was excluded from this study because examination by qPCR showed for these genes highly variable transcript levels caused by a transfer of small amounts of lens epithelium during retina isolation [35].

### Web tools

Gene annotation of the genes on the array was improved using web-based annotation tools DAVID-2006 (National Institute of Allergy and Infectious Diseases (NIAID), NIH) [36] and Source (Stanford University, Stanford, CA). Of the 20,280 features, 92% could be linked to a UniGene Cluster ID; representing 14,127 unique UniGenes. Gene Ontology (GO) analysis used to identify overrepresented or depleted functional groups of genes and was performed with GOstat [37] and DAVID-2006 [36]. Lists of selected genes-of-interest were compared with the total set of genes present on the array. Fisher's Exact Test was performed to rank GO-terms in *GO*stat. The enrichment calculation performed in DAVID, called EASE score, is a conservative adjustment to the Fisher exact probability that weighs significance in favor of themes supported by more genes [36,38,39]. The derived P-values are a score system to organize the view and to facilitate exploration rather than a strict decision making line; scores p<0.01 were considered to be of potential interest.

### Real time quantitative polymerase chain reaction

For qPCR, 2 μg total RNA from each individual retina was dissolved in 4 μl H_2_O, DNase I treated (0.5 unit DNAseI, Amplification Grade, Invitrogen). The RNA sample was reverse transcribed into first strand cDNA with 100 U Superscript III Reverse Transcriptase (Invitrogen) and 50 ng random hexamer primers. The resulting cDNA sample served as a template for SYBR® green RT-qPCR analysis (ABI 7300 Real-time system; Applied Biosystems, Nieuwerkerk a/d lJssel, The Netherlands) using SYBR® Green PCR Master Mix (Applied Biosystems Nieuwerkerk a/d lJssel, The Netherlands). Sequences of the used primers are available upon request. The amplification efficiencies had values close to 2 for all primer combinations. The resulting C_t_ values were converted to absolute amounts of cDNA present in the sample (E*^-C^*^t^) [28]. To correct for differences in cDNA load between the different samples, we normalized the target PCR to a set of reference PCRs. For this, qPCR data for a selection of candidate reference genes on experimental and control samples was used as input for a geNorm analysis [28,40]. Transcript levels of *Rho* (rhodopsin), *Hprt* (hypoxanthine phosphoribosyl transferase), *Nef3* (neurofilament 3), *Thy1* (thymus cell antigen 1, θ), and *Prkca* (protein kinase Cα) were most stable and used for normalization.

## Results

### Histology

**Figure 1:**
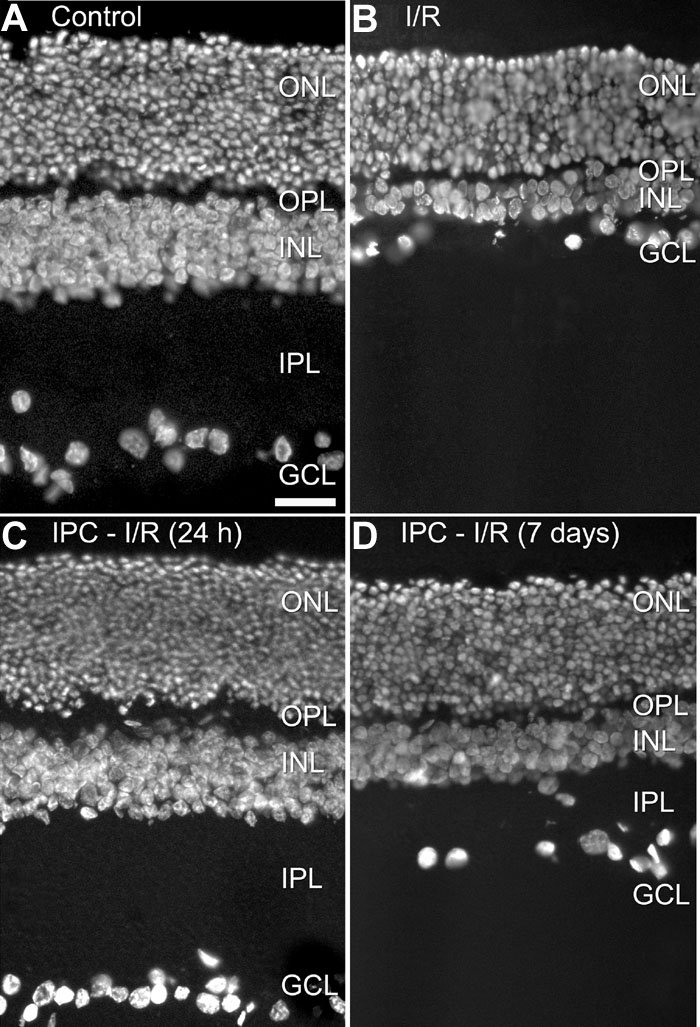
Sections of rat retina stained with the fluorescent DNA-stain 4,6 diamidino 2 phenylindole (DAPI). **A**: Control, retina of a sham-treated eye. **B**: I/R, retina 7 days of reperfusion after 60 min of ischemia. Note the decrease in thickness of INL and IPL, and the reduced number of somata in the ganglion cell layer. **C**: IPC-I/R (24 h), retina preconditioned by 5 min of ischemia, followed 24 h later by 60 min of ischemia and sacrificed 7 days later. No histological changes can be found. **D**: IPC-I/R (7 days), retina preconditioned by 5 min of ischemia, followed 7 days later by 60 min of ischemia and sacrificed 7 days later. The effect of preconditioning has diminished, but the loss of IPL thickness is still partially prevented. ONL represents outer nuclear layer, OPL represents outer plexiform layer, INL represents inner nuclear layer, IPL represents inner plexiform layer, GCL represents ganglion cell layer. All images were taken at the same magnification. Scale bar in **A** represents 25 μm.

To study the effects of IPC, 5 min of ischemia was induced and followed, after various reperfusion intervals (24 h, 48 h, 7 days), by a full 60 min ischemic insult. Seven days later, when degeneration has taken its full course, the retinas were examined using histology. In not-conditioned animals, retinal ischemia for 60-min leads to a significant reduction in the number of neurons from the ganglion cell layer (GCL) to 63±11% and to a thinning of the inner plexiform layer (IPL) to 39±5% (untreated control retinas set at 100%; n=4); mean±SEM [25]. Animals that were challenged 24 h after induction of tolerance (IPL, 100±8%; GCL, 100±7%; n=3) were morphologically indistinguishable from a large group of controls (IPL, 100±2%; GCL, 100±5%; n=31), demonstrating complete protection. This is illustrated in Figure 1. At 48 h, a minor reduction in IPL (82±18%) and GCL cell count (88±8%) was found. Seven days after induction of tolerance, the protective effect was lost for GCL cell count (50±9%) but a mild protective effect persisted for IPL thickness (60±11%; Figure 1). These results are in agreement with the published findings on the time course of transient tolerance induction in mouse and rat retina [10,11]. Based on these results, it was decided to study the changes in gene expression during the build-up of tolerance (reperfusion intervals: 1, 3, 6, and, 12 h), at fully established tolerance (24 and 48 h) and after tolerance had faded (7 days).

### Microarray-based identification of genes of interest

The microarray analysis here must be regarded as a screening tool to select potential genes of interest, to be used as starting point for Gene Ontology analysis and confirmatory qPCRs on the individual animals underlying the pooled samples. The following criteria were applied to the microarray data to select for features-of-interest (see Methods section for details): (1) a fold change of >1.7, at least at one time point and, (2) a methodological error of p<0.01, assigned by the normalization software, to exclude features with a low signal to noise ratio. The number of selected features for each experimental condition is presented in Table 1. The list includes a total of 134 different features. The 60-mer probe sequences were all blasted against GenBank, and only those features with a perfect homology to well annotated genes were used for further study, resulting in 104 genes-of-interest. Comparison of the different time points showed some overlap in the changes in gene expression profile: 18 genes were independently selected at two different reperfusion times, six at three, and one gene (*Anxa1*) at four different time points.

**Table 1:** Number of selected features-of-interest for each experimental condition.

**Title**	**Signatures**	**Up**	**Down**
IPC 1 h	46	34	12
IPC 3 h	34	15	19
IPC 6 h	30	20	10
IPC 12 h	13	12	1
IPC 24 h	21	3	18
IPC 48 h	28	7	21
IPC 7 days	10	7	3

A feature is defined to be of interest when having a dye log ratio corresponding to a difference of >1.7 fold (up or down) and an assigned technical error of p<0.01.

### Gene ontology term analysis

The list of 104 UniGene cluster IDs of the selected genes-of-interest was subjected to GO analysis to identify functions/processes of interest [37]. Using *GO*stat-rat genome database, 68 of the 104 genes (65%) were associated with one or more GO-terms. Using DAVID-2006, 90 genes (85%) were associated with one of more GO-terms by using different annotation sources. The statistically most significant result from both tools is an overrepresentation of GO-terms for (1) aminoacyl-tRNA synthetase activity, (2) translation, (3) amino acid metabolism and transport, (4) cell death, and (5) transcription, (6) nuclear lamina, and (7) anchored to plasma membrane (Table 2). The "Functional annotation clustering" tool of DAVID with high stringency classification settings confirmed these gene clusters except for nuclear lamina and anchored to plasma membrane. A summary is given in Table 3, the complete set is presented in Appendix 2. The GO-term analysis was also carried out for each reperfusion interval and identified a significant overrepresentation of genes associated with transcription at 1 h (p<0.0004), aminoacyl-tRNA synthetases at 24 and 48 h (p<5.4.10^-8^ and p<4.2.10^-7^), and amino acid metabolism at 48 h (p<5.2.10^-5^) and amino acid transport at 48 h (p<9.3.10^-5^). Repeating the GO-term analysis on the list of genes based on less stringent selection settings (>1.5 fold, p<0.01; 163 genes) yielded comparable overrepresented GO-terms.

**Table 2:** Gene ontology results for the changes in gene expression after ischemic preconditioning.

**GO-terms**	**Genes**	**C/T**	**GO**	**p**
Aminoacyl-tRNA synthetase activity	Yars, Tars, Gars, Iars	4/22	GOs	0.0001
Aminoacyl-tRNA synthetase activity	Yars, Tars, Gars, Cars, Lars	5	DaV	0.0003
Translation	Yars, Tars, Iars, Bzw2, Gtpbp2, Eif2s2	6/77	GOs	0.0002
Translation	Yars, Tars, Gars, Cars, Lars, Bzw2, Gtpbp2, Eif2s2	8	DaV	0.0001
Amino acid metabolism	Yars, Tars, Iars, Asns, Slc6a6, Slc7a1	6/130	GOs	0.0038
Amino acid metabolism	Yars, Tars, Gars, Cars, Lars, Asns, Slc6a6, Slc7a1	8	DaV	0.0021
Amino acid transport	Slc3a2, Slc6a6, Slc7a1	3/29	GOs	0.0044
Amino acid transport	Slc3a2, Slc6a6, Slc7a1, Slc38a2	4	DaV	0.0064
Cell death	Emp1, Anxa1, Trib3, Lcn2, Nr4a1, Klf10, Lgals7, Yars, Ccl6, Fosl2	10/382	GOs	0.0119
Cell death	Emp1, Anxa1, Trib3, Lcn2, Nr4a1, Klf10, Lgals7, Yars, Ccl6, Perp	10	DaV	0.0092
Transcription	Atf3, Trib3, Nr4a1, Nr4a3, Cebpd, Klf10, Egr1, Egr4, Jund, Fosl2, Fgfbp1, Snf1lk, Zfp36, Fos, Cdc73	15/600	GOs	0.0024
Transcription	Atf3, Trib3, Nr4a1, Nr4a3, Cebpd, Klf10, Egr1, Egr4, Jund, Fgfbp1, Snf1lk, Zfp36, Arid1a, Klf4, Atf7ip	15	DaV	0.0160
Nuclear lamina	Emd, Lmna	2/6	GOs	0.0019
Anchored to plasma membrane	Cp,Tmprss8	2/13	GOs	0.0095

C/T indicates number of signatures genes assigned to GO-term/total number of genes on the array assigned to GO-term (GOstat only), GO indicates analysis tool, GOs indicates GOstat, DaV indicates David, p indicates Fisher exact p-value.

**Table 3:** Results of gene ontology-term based clustering.

**UniGene name**	**1 h**	**3 h**	**6 h**	**12 h**	**24 h**	**48 h**	**7 d**	PCR	Abbreviated description
**Aminoacyl-tRNA synthetase, [David Enrichment score 3.59], translation**									
Tars	-1.1	1.3	-1.3	1.1	-1.7	-1.5	1	=	Rat threonyl-tRNA synthetase
Cars	-1.2	1.2	-1.2	-1.1	-1.8	-1.4	1		Cysteinyl-tRNA synthetase
Lars (prb1)	-1.2	-1	-1.1	1.1	-1.7	-1.7	-1.1	=	Leucyl-tRNA synthetase
Lars (prb2)	-1.3	1	-1.1	1.1	-1.7	-1.6	-1	=	Leucyl-tRNA synthetase
Yars	-1	1.1	-1.1	-1.1	-1.7	-1.5	-1	=	Tyrosyl-tRNA synthetase
Gars	-1.2	-1	-1.1	1	-1.7	-1.4	1		Glycyl-tRNA synthetase
Iars (prb1)	-1.1	1.1	-1.2	1	-1.8	-1.6	-1	=	Isoleucine-tRNA synthetase
Iars (prb2)	-1.3	1.3	-1.2	-1.1	-1.8	-1.5	1.1	=	Isoleucine-tRNA synthetase
Bzw2	-1.1	-1	-2.3	1.1	-1.1	-1.3	1.2		Basic leucine zipper and W2 domains 2
Gtpbp2	-1.2	1	-1.1	-1.1	1	-2.4	1		GTP binding protein 2
Eif2s2	-1.3	1.2	-1.2	1.1	-1.4	-1.7	1	=	"Rat eukaryotic translation initiation factor 2, subunit 2 beta"
Mthfd*	1.2	1	-2.4	-1	-2.3	-2.8	-1.2		Similar to methylenetetrahydrofolate dehydrogenase (NAD)
Pop5*	1.4	1	-2.9	-1.1	1.3	1.6	1.8		"Processing of precursor 5, ribonuclease P/MRP family"
Asns*	-1	1.1	-1.1	-1	-1.7	-1.5	-1	=	Asparagine synthetase
**Amino acid transport [David Enrichment score 1.82]**									
Slc3a2	-1.1	1.2	-1.4	-1.1	-1.8	-1.4	-1.1	=	"Activators of dibasic and neutral amino acid transpor, member 2"
Slc6a6	1.7	-1	1.3	-1.4	1.5	1.3	1.2	=	"Neurotransmitter transporter, taurine, member 6"
Slc7a1	-1.1	1.4	-1.6	-1.1	-2.7	-2.2	-1.1	=	"Cationic amino acid transporter, y+ system, member 1"
Slc38a2	-1.2	1	-1.5	1	-1.5	-1.7	-1.1	=	"Solute carrier family 38, member 2"
**Cell death [David Enrichment score 1.88]**									
Emp1	-1.5	-1.2	3.5	1.9	-1.1	-1.5	-1.3		Epithelial membrane protein 1
Anxa1	-1.7	-2.3	3.8	2.1	1.1	-1.2	-1.1	=	Annexin A1
Trib3	-1.1	-1.2	-2.9	-1.2	-1.3	-1.9	-1	=	Tribbles homolog 3 (Drosophila)
Lcn2	1.2	1.2	-1.6	2.3	2.5	-2	-1.6		Lipocalin 2
Perp1	-1.3	-1.7	2.3	1.2	-1.2	-1.1	-1.2		"PERP, TP53 apoptosis effector"
Lgals7	-1.1	-2.7	2.9	-1.2	1	-1	-1.3		"Lectin, galactose binding, soluble 7"
Ccl6	-1.5	-1.6	2.2	1.2	-1	-1.1	-1.2		Chemokine (C-C motif) ligand 6
Klf10	2	-1.1	1.1	1.1	1	-1.1	-1		Kruppel-like factor 10
**Regulation of transcription, DNA-dependent [David Enrichment score 1.55]**									
Atf3	-1.1	-1.3	-3.1	1.1	-2	-3.3	-1.2	=	Activating transcription factor 3
Nr4a1	4	-1.4	-1.3	-1.2	1	-1.3	1	=	"Nuclear receptor subfamily 4, group A, member 1"
Nr4a3	5.4	-2.4	-2.1	1	-1.2	-1.9	-1.8	=	"Nuclear receptor subfamily 4, group A, member 3"
Cebpd	1.5	1.2	1.2	2	1.5	-1.9	-1	=	"CCAAT/enhancer binding protein (C/EBP), delta"
Egr1	5	1.2	3.1	-1.1	-1.5	-2.1	-1.1	=	Early growth response 1
Egr4	2.9	-1.4	-1.1	-1.2	-1.1	1.1	-1.2		Early growth response 4
Jund	1.7	-1	-1.1	-1.1	1.1	-1	-1		Jun D proto-oncogene
Fgfbp1	-1.2	-1.3	1.5	1.1	-1.1	-2	-1		Fibroblast growth factor binding protein 1
Snf1lk	1.2	-2	-1.4	-1.1	-1	-1.2	-1.2		SNF1-like kinase
Zfp36	1.8	1.1	1.1	1.1	-1.2	-1.2	1.1		Zinc finger protein 36
Arid1a	1.2	1.4	1.2	1.4	1.4	2.7	1.5		AT rich interactive domain 1A (Swi1 like)
Klf4	2.1	-1	-1.3	1.2	-1.1	-1.3	-1.2		Kruppel-like factor 4 (gut)
Atf7ip	-1.1	-1.1	-2.7	1.1	-1.6	-1.8	-1.9		Activating transcription factor 7 interacting protein
c-fos	2.6	-1.4	1	1.1	-1.1	-1.3	1	=	FBJ murine osteosarcoma viral oncogene homolog (Fos)
Cdc73	-1.2	-2.3	-1	1.2	1.2	1.1	-1.1		"Cell division cycle 73, Paf1/RNA polymerase II complex component"
Fosl2*	1.2	-1.7	-1.2	1	-1.1	-1.7	-1.3		Fos-like antigen 2
Hes5*	-1.7	1	1.2	-1.1	-1	1	1.1		Hairy and enhancer of split 5 (Drosophila)
Arid2*	1.2	-1.2	1.1	1	1.1	-2.3	-1.3		AT rich interactive domain 2 (Arid-rfx like)
Arid5a*	1.8	-1.2	-1.2	-1	-1.1	-1.1	-1.1		AT rich interactive domain 5A (Mrf1 like)
Nab2*	2.3	1.8	1.3	-1	1.2	1	1.1	=	Ngfi-A binding protein 2
Lix1*	1.3	2.1	1	-1.2	1	1.3	1.1		Similar to Lix1 homolog (mouse) like (LOC499677)
Phf20*	1.3	1.7	-1	-1.2	1.2	1	-1.1		Similar to PHD finger protein 20
**Anchored to cell membrane**									
Cp	-1.2	1	1.2	2	1.2	-1.2	1.2		Ceruloplasmin
Tmprss8	-2.2	-1.8	1.2	1.2	1.2	1.1	1.3		"Transmembrane protease, serine 8 (intestinal)"
**Intermediate filament, cytoskeletal part**									
Lmna*	1.7	1.3	-1.1	-1	1	-1	-1		Lamin A
Gfap*	1.2	1.6	1.4	2	1.8	-1.4	1.3		Glial fibrillary acidic protein
Emd*	1.7	1.2	1.1	-1.2	-1.1	-1	-1		Emerin
Tubb6*	1.6	1.7	1.8	1.2	1.2	-1.5	-1		"Tubulin, beta 6"
Pdlim3*	1.8	1.7	1.2	1.2	1.1	1	1.2		PDZ and LIM domain 3
Dsc2*	-1.3	1.4	2.9	1.4	-1	-1.2	-1.1		Desmocollin 2
Mpp2*	1.1	-1.1	1.2	2.9	1.1	1.5	1.4		"Membrane protein, palmitoylated 2"
Enc1*	1.8	1.2	1.9	-1.5	1.7	2.2	3.6		Ectodermal-neural cortex 1
Nexn*	-1.2	-1	-1	1	-1.7	-1.1	1.1		Nexilin
Mfap3*	-1.1	2.4	1.1	-1.1	1.4	-1	1.4		Microfibrillar-associated protein 3
Sdc1*	-1.1	-1.8	2.4	1.1	-1.6	-2	-2.4		Syndecan 1

Fold change for the different ischemic preconditioning intervals are in red color when fold change is least 1.7-times and the methodological error is p<0.01, in black when the methodological error is p<0.01. PCR column indicates whether the qPCR assay confirms the array outcome (=). UniGene names indicated by asterisk are added to the cluster on basis of other functional annotation than GOStat or DAVID. Lars and Iars were represented by two different probes (prb) on the array.

### Quantitative polymerase chain reaction

RT-qPCR assays for 21 different members from the 98 genes-of-interest were carried out on the individual retinal cDNA samples (Table 3 and Appendix 3 for details). The average coefficient of variation for the 21 genes at the different reperfusion groups was 47±11%, confirming our previous findings on the variation of expression levels [26,28] and supporting the applied selection criteria for genes-of-interest selection. Among the transcripts used for normalization were *Nef3* and *Thy1*, two established markers for neurodegeneration after full ischemia in the retina [26,28]. The transcript level stability of these genes demonstrates the absence of neurodegeneration after IPC.

**Figure 2:**
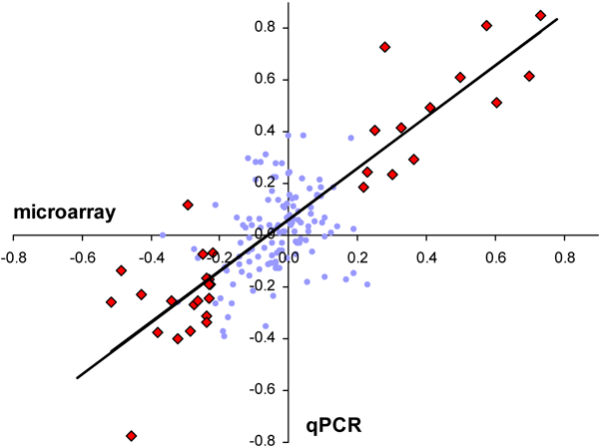
Linear regression between microarray and qPCR results both expressed as log_10_ fold-change for different experimental groups with reference to the levels in controls. Each of the 161 data point shown represents the average of five or six animals; R^2^=0.54, y=0.85+0.04 (blue dots and red diamonds). The trend line shown is based on the subset of microarray data points meeting the selection criteria (>1.7 fold and p<0.01; n=34) related to 21 different genes-of-interest; R^2^=0.85, y=0.99+0.06 (red diamond symbol).

For each experimental condition, the average change in transcript level compared to controls was calculated as log_10_ experimental/control and plotted against the log_10_ values derived from the microarrays. The result of the linear regression is presented in Figure 2. The correlation coefficient for all 161 data points: R^2^=0.54. For data points meeting the microarray selection criteria (>1.7 fold and p<0.01; n=35) R^2^=0.84.

### Aminoacyl-tRNA synthetases

The microarrays revealed for six different aminoacyl-tRNA synthetases (ARSs) encoding genes (*Iars, Lars*, *Cars*, *Yars*, *Gars*, *Tars*) reduced expression levels by 30-45% at 24 and 48 h after IPC induction. The transcript levels of *Iars*, *Lars*, *Yars*, and *Tars* were investigated by qPCR and the average reduction was 31±7% at 24 h and 49±3% at 48 h. Of the 21 ARSs known to exist, 14 were represented by probes on the array. Examination of these features revealed reduced transcript levels also for *Sars1*, *Sars2*, *Nars*, and *Farsla* at 24 and 48 h after IPC, although the degree of change was less prominent with 20-25% reduction. For *Vars*, *Quars*, *Wars*, *Dars*, and *Kars*, no changes greater than 10% were detected. In summary, nine out of the 14 studied ARSs showed reduced transcript levels at 24 and 48 h after IPC induction. In addition, several members of the solute carrier family, encoding for amino acid transporters (*Slc3a2*, *Slc7a1*, *Slc38a2*), and the initiation factor *Eif2s2* followed the same pattern of changes with a reduction of 30 and 60% at 24 and 48 h IPC, respectively. Comparable changes were found by qPCR.

### Heat shock proteins

The transcript level of *Hsp1b*, also known as *Hsp27*, has been reported to be upregulated after IPC induction, possibly as a consequence of an increased Hif1a expression [10,14-16]. No significant changes were found for *Hif1a* transcript levels either by microarray or by two different qPCR assays. *Hspb1* was not present on the array design; it was therefore studied by qPCR only. After IPC induction, *Hsp1b* transcript levels peaked at 3 h IPC (457%) with lesser increased levels thereafter (139% at 6 h, 133% at 12 h, 93% at 24 h). *Hmox1* (*HO-1*, *Hsp32*) is an extremely sensitive detector of ischemia [10]. Microarrays showed an increase of 30% at 1, 3, and 6 h, while qPCR revealed a more robust significant increase of 40%, 130%, and 87% at 1, 3, and 6 h.

Both microarray and qPCR did not detect IPC-induced changes in *Hspa1a* (*Hsp70*) or *Hspca* (*Hsp90*) transcript levels.

## Discussion

A short period of retinal ischemia establishes a temporal but robust form of neuroprotection against a subsequent, otherwise damaging, ischemic insult [10,11,16-19]. This form of neuroprotection, termed IPC or tolerance, has been observed in several neuronal and non-neuronal tissues [13,22-24,41]. We conducted a series of microarray assays to study alterations in the retinal gene expression profile after IPC in order to gain insight in the response at the transcriptome level that results in the neuroprotective state. Our knowledge of quantification of transcript levels in control and ischemic retinas was used to formulate selection criteria to identify features-of-interest. Changes in transcript level of at least 1.7 fold were found for 134 of the 20281 features present on the array at any time after IPC, with a maximum of 46 features at 1 h after preconditioning. Not all feature probes could unquestionably be linked to genes with a proper annotation in rat genomic databases. This reduced the list to 104 genes-of-interest. The GO-term analysis on this set of genes identified a significant overrepresentation of ARS activity, translation, amino acid metabolism and transport, transcription factors, and cell death.

### Aminoacyl-tRNA synthetases

The reduced transcript levels of nine different ARSs and of genes involved in amino acid metabolism and transport was restricted to 24 h and 48 h after IPC, suggesting that these changes may be an effector mechanism of the neuroprotective state. Some of the downregulated ARSs form a macromolecular complex with subunits of elongation factor 1 (Lars, Fars, Gars). Furthermore, Iars and Lars are integral parts of a multi-ARS complex [42,43]. The precise function of this molecular assembly and the functional roles of components of the multi-ARS complex are not well understood. However, a reduced expression of ARSs, in concert with a reduction in expression of amino acid transporters, may lead to diminished efficiency of protein translation. In addition, expression of *Eif2s2* (eukaryotic translation initiation factor 2, subunit 2/β; Eif2b) was decreased at the same time. Eif2b is a key limiting factor in the regulation of protein synthesis and has been linked to cell survival [44,45]. Because the decreased transcript levels are limited to 24-48 h after IPC induction, a reduction of protein synthesis is correlated with observations that a de novo protein synthesis is required for the establishment of successful IPC [10,11,18]. Inhibition of protein synthesis by cycloheximide reduced ischemic damage in brain [46] and spinal cord [47,48], showing that suppression of protein synthesis can be beneficial for cell survival. How an overall down-regulation of the rate of protein synthesis could mediate effective neuroprotection is not clear, but it may attenuate the synthesis of proteins that cause ischemia-mediated cell loss.

### Transcription factors

Changes in the expression of transcription factors in response to IPC is a general finding in several models [23,24,49]. The GO-term "regulation of transcription" (15 genes) was significantly overrepresented at 1 h after the induction of IPC. The response of *Egr1* (*Ngfi-A*, *Krox-24*, *zif268*), *Nr4a1* (*Ngfi-B*, *Nur77*), *Egr4* (*Ngfi-C*) and the increase of *Nab2* (Ngfi-A binding protein 2) may be the result of an increased nerve growth factor release, as has been observed after rat brain IPC [50,51].

Evidence for a role of Hif1 in brain and retinal preconditioning has been presented [12,14,52]. Our results however showed no upregulation of *Hif1a* or of its heterodimerization partner *Hif1b* (*Arnt*) after IPC. qPCR assays with two different primer sets confirmed this observation. Moreover, except for *Hsp27*, none of the *Hif1*-dependent genes that are increased after hypoxic brain conditioning (*Epo*, *Fgf2*, and *Vegf*) were found to be altered in our study [12,13,52]. Our data are not in support of Hif1a as an essential mediator of retinal IPC [12,14]. The finding that Hif1a-deficient mice were protected from hypoxia-induced cell death suggests that activation of Hif1a could even be deleterious in vivo [53].

A class of transcription factors commonly implicated in IPC are Heat Shock Proteins (Hsps) [15,16,23,24,49,54,55]. Retinal IPC increased gene expression of *Hspb1* (*Hsp27*) but not of *Hspa1a* (*Hsp70*) or *Hspca* (*Hsp90*) or any other Hsp encoding genes on the arrays [15]. Our results are therefore in good agreement with these findings and do not support a dominant role of Hsps in retinal IPC. *Hmox1* (Heme oxygenase (decycling) 1, *Hsp32*) has a cytoprotective effect in many tissues by attenuating the overall production of reactive oxygen species [56]. Roth et al. reported the upregulation of Hmox1 protein levels at 24-72 h after retinal IPC [10]. In the model of cortical spreading depression *Hmox1* was increased at 24 h (2 fold) [49]. In line with these findings, both microarrays and qPCR showed increased *Hmox1* transcript levels up to 6 h after IPC, suggesting a protective role of Hmox1 also in the retina.

### Cell death

The overrepresentation of genes associated with cell death indicates that an interference of pathways leading to cell loss is a target of IPC. Changes in the expression of *Anxa1* (annexin I) and of *Trib3* (tribbles homolog 3) have not yet been associated with IPC. Anxa1 belongs to a family of Ca^2+^-dependent phospholipid binding proteins, and although it was originally described as a phospholipase A2-inhibitory protein, Anxa1 affects various other components of inflammatory responses such as the inhibition of firm adhesion of leucocytes to endothelium [57,58]. The upregulation of *Anxa1* at 6 and 12 h may therefore have an anti-inflammatory effect and underlie the IPC-dependent prevention of the increase in the number of rolling leukocytes observed after ischemia [19,59]. The downregulation of *Trib3* may also play a role in cell fate in response to environmental challenges by interacting with apoptosis promoting transcription factors Atf4 and Chop (Ddit3) [60]. Knockdown of Trib3 expression decreased endoplasmatic reticulum (ER) stress-dependent cell death [61].

### Microarray results of ischemic preconditioning-induced gene expression changes in other ischemic models

Given the fact that IPC is not unique for the retina, it is of potential interest to compare our findings with the outcome of microarray studies on preconditioning in other neuronal tissues. However, it must a priori be pointed out that the sensitivity for ischemic damage differs greatly among neuronal tissues. The retina is relatively insensitive to ischemic damage with cell loss resulting only after 45 min of ischemia, while IPC can be established by just 5 min of ischemia resulting in full protection. In other tissues, the duration of the ischemic episode leading to either IPC or damage is more similar. In these tissues induction of IPC will also lead to some degree of cell loss and, consequently, IPC-evoked mechanisms are obscured by damage-related changes. Compared to other neuronal tissues, differentially expressed genes after retinal IPC are therefore less prone to be a mix of changes leading to preconditioning and neurodegeneration. Four studies were reviewed in detail. (1) IPC in the spinal cord is evoked by 3 min of ischemia while damage and functional loss occurs already after 10 min [23]. Moreover, spinal cord IPC is not fully protective against the loss of motor function and number of spinal neurons [54]. Microarray analysis of spinal cord IPC identified changes in *Hspa1a*, *Mt1a* (metallothionein 1a), *Egr1* (early growth response 1), and *Dusp1* (dual specificity phosphatase 1) [23]. Our results show only the transient upregulation of *Egr1* whereas *Hspa1a* and *Ptpn16* (*Dusp1*) were not significantly altered, and *Mt1a* was not represented by specific probes on our array. (2) The hippocampal CA1 region is prone to ischemic damage by periods as short as 6 min leading to a loss of 73% of pyramidal neurons [24]. Although 2 min of ischemia leads to protection against a full ischemic insult given three days later, a cell loss of 20% can not be prevented. The induction of hippocampal IPC was associated with altered expression of 96 features in the CA1 region [24], including 13 transcription factors of which *c-fos*, *Egr1*, *Egr4*, *Klf10*, *Nr4a1* are also found in the present study. (3) Hypoxic preconditioning (1 or 6 h 8% O_2_) has a neuroprotective effect on focal cerebral ischemia in mice. Again, the protection is only partial, reducing the infarct volume by about 30% [52]. Microarray based screening identified a differential expression for 21 genes, including 3 genes identified in the present study (*Atf3*, *Gfap*, *Cebpd*) [13]. (4) In rat brain, preconditioning by 10 min of transient middle cerebral artery occlusion reduced the infarct volume by 38% resulting from 1 h occlusion [55]. In total 32 genes were found to be differentially expressed after preconditioning. Only *Hspb1* (*Hsp27*) was also identified by our study.

In conclusion, there is little overlap in the set of genes found in our study, and the sets found in other preconditioning models, which may be attributed to the confounding effects of neurodegenerative processes taking place in parallel with the establishment of tolerance. Interestingly, cortical spreading depression can act as a preconditioning trigger without causing degeneration. A microarray study on rat cortex isolated after an average of 7 spreading depressions applied over a period of 2 h identified 353 regulated genes [49]. A cluster of 36 immediate early genes and transcription factors were upregulated at 3 h, of which *Fosl2*, *c-fos*, *Egr1*, *Egr4*, *Atf3*, *Nr4a1*, *Nr4a3*, and *Zfp36* were also identified in our study, suggesting possible common pathways leading to protection.

Our microarray study on the changes in gene expression profile after retinal preconditioning has resulted in evidence that a restriction of protein translation rate may be a potential effector of neuroprotection. Further research is needed to substantiate this hypothesis in order to evaluate the protective effects of protein synthesis inhibition to prevent ganglion cell loss in experimental models.

## Appendix 1. Microarray analyses.

The complete list of all signatures, log_10_ ratio, log ratio error, and p-values for the genes assessed in this report is provided as supplemental data in a compressed Excel file. To access these data, click or select the words "Appendix 1". This will initiate the download of a compressed (zip) archive that contains an Excel file. This file should be uncompressed with an appropriate program (the particular program will depend on your operating system).

## Appendix 2. Clustering data.

The complete set of gene clustering data is provided as supplemental data in a compressed Excel file. To access these data, click or select the words "Appendix 2". This will initiate the download of a compressed (zip) archive that contains an Excel file. This file should be uncompressed with an appropriate program (the particular program will depend on your operating system).

## Appendix 3. Quantitative PCR validation.

RT-qPCR assays for 21 different members from the 98 genes-of-interest were carried out on the individual retinal cDNA samples. Complete analysis of this is provided as supplemental data in a compressed Excel file. To access these data, click or select the words "Appendix 3". This will initiate the download of a compressed (zip) archive that contains an Excel file. This file should be uncompressed with an appropriate program (the particular program will depend on your operating system).
